# Percutaneous treatment of severe branch pulmonary stenosis in children who underwent branch pulmonary artery stenting in infancy: how to deal with stents that cannot be expanded to adult size—case reports

**DOI:** 10.1093/ehjcr/ytag156

**Published:** 2026-03-05

**Authors:** Ines Hribernik, Kerstin Trociewicz, Daniela Kiski, Philippe Grieshaber, Andreas Brünen, Alexander Schnabel, Fabian Rohlfing, Matthias Sigler

**Affiliations:** Department of Paediatric Cardiology, University Hospital Münster, Albert-Schweitzer-Campus 1, 48149 Münster, Germany; Department of Paediatric Cardiology, University Hospital Münster, Albert-Schweitzer-Campus 1, 48149 Münster, Germany; Department of Paediatric Cardiology, University Hospital Münster, Albert-Schweitzer-Campus 1, 48149 Münster, Germany; Divison of Paediatric Cardiac Surgery, University Hospital Münster, Albert-Schweitzer-Campus 1, 48149 Münster, Germany; Department of Anaesthesiology, Intensive Care and Pain Medicine, University Hospital Münster 1, 48149 Albert-Schweitzer-Campus, Münster, Germany; Department of Anaesthesiology, Intensive Care and Pain Medicine, University Hospital Münster 1, 48149 Albert-Schweitzer-Campus, Münster, Germany; Department of Anaesthesiology, Intensive Care and Pain Medicine, University Hospital Münster 1, 48149 Albert-Schweitzer-Campus, Münster, Germany; Department of Paediatric Cardiology, University Hospital Münster, Albert-Schweitzer-Campus 1, 48149 Münster, Germany

**Keywords:** Infantile branch pulmonary artery stenting, Intentional stent fracture and re-stenting, Case report

## Abstract

**Background:**

Current paediatric and congenital interventional cardiologists are increasingly dealing with patients who underwent branch pulmonary artery stenting during infancy and present with severe re-stenosis at follow-up due to reaching the limit of stent expansion because stent dimensions no longer match the distal vessel diameter.

**Case summary:**

We report two cases of older children who underwent branch pulmonary artery stenting in infancy where percutaneous re-stenting with adult-sized stents relieved the stenosis completely and has been employed as a lifelong strategy.

**Discussion:**

Serial balloon angioplasty with high-pressure balloons, planned re-stenting with adult-sized stents before intentional fracture of the initially implanted stents, and choosing the right timing and types of stents are parts of the treatment strategy that can be implemented successfully in many paediatric patients with branch pulmonary artery stenosis.

Learning points

- Lifelong treatment strategy is required for successful percutaneous management of branch pulmonary artery stenosis in small infants and children.
- Serial stent balloon angioplasty with incremental balloon sizes has to be undertaken in order to compensate for somatic growth throughout childhood.
- Planned re-stenting with adult-sized stents when approaching the maximal stent expansion diameters, followed by intentional fracture of the initial stent with high-pressure balloons, is highly advisable.

## Introduction

Treating branch pulmonary stenosis in infants and small children remains a lifelong challenge for patient management; although small size peripheral vascular, renal, and biliary stents [e.g. Formula (Cook Medical, IN, USA) and Valeo (Bard Medical, NJ, USA)] are being used successfully for relieving obstruction in small pulmonary arteries, the limited stent expandability remains a concern where significant patient somatic growth is to be expected. Although new materials are coming onto the market, such as stents with predetermined breaking points in order to help with future upsizing (for example, Bentley® BeGrow™ stent)^1^ or absorbable metal stents,^2^ which will likely change the prospect for our small patients, the current paediatric and congenital interventional cardiologists are now increasingly dealing with patients who underwent branch pulmonary artery (PA) stenting as infants and present with severe branch PA stenosis in the follow-up due to reaching the limit of stent expansion that no longer matches the distal vessel diameter.

Case series and studies have been reported regarding redilatation of endovascular stents in congenital heart disease,^3,4^ as well as the possibility and safety of the intentional stent breakage.^5^ However, patients with persisting branch PA stenosis even after maximal stent expansion and/or breakage remain an ongoing concern.

We report two cases of successful branch PA stenosis relief after intentional stent fracture and percutaneous re-stenting a decade after infantile branch PA stent implantation and discuss lessons to be learnt.

## Summary figure

**Figure:**
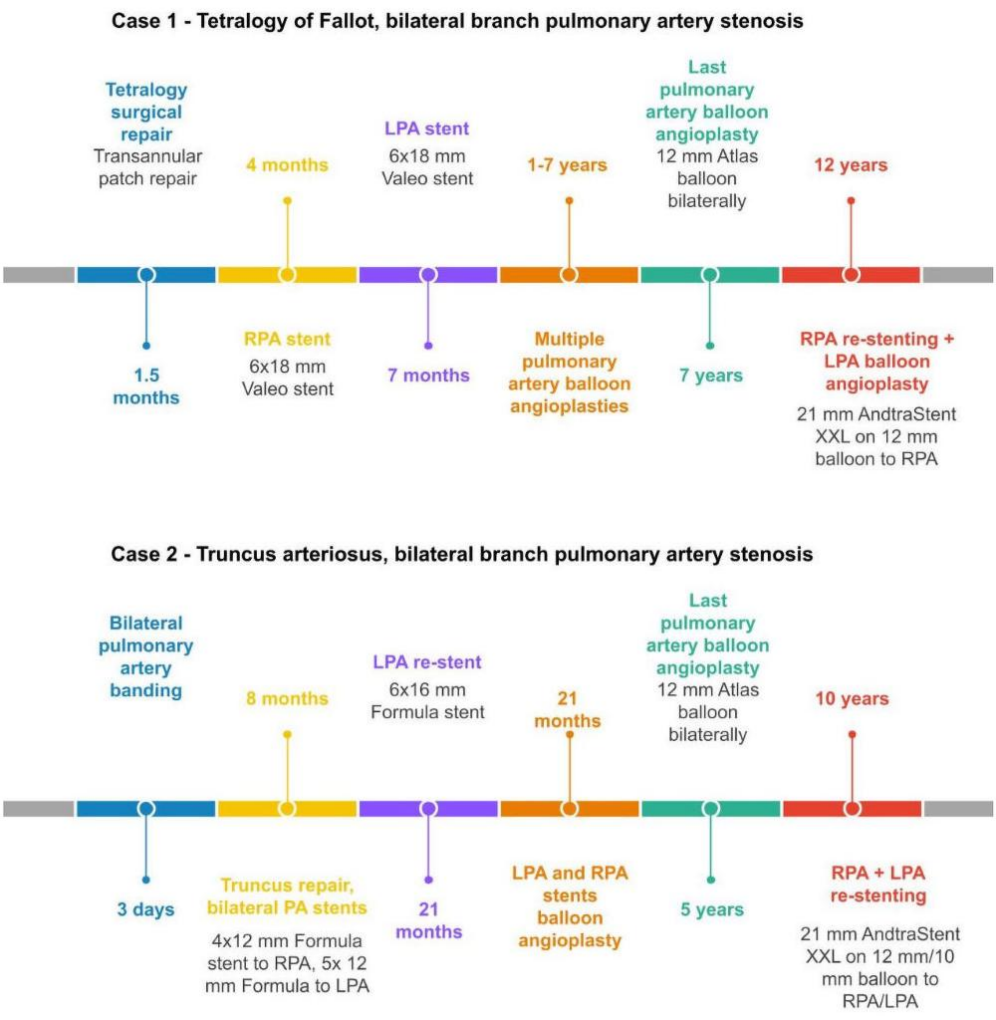


## Case presentation

### Case 1

The first case is a 12-year-old Caucasian boy, born with tetralogy of Fallot. A full surgical transannular patch repair occurred early at the age of 1.5 months due to cyanotic spells. Both right pulmonary artery (RPA) and left pulmonary artery (LPA) were stented 2 and 5 months post-surgery, respectively, with 6 × 18 mm Valeo stents. Multiple sequential balloon angioplasties of the stents had to be performed during follow-up, with the last dilation at the age of 7 years, where both stents were dilated with high-pressure balloons with the final balloon size of 12 mm Atlas (Bard Medical, NJ, USA) to 18 atmospheres (atm).

**Figure 1:**
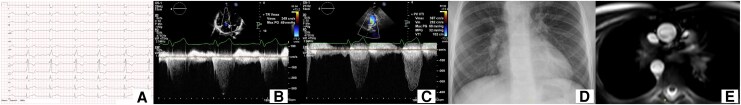
Case 1 pre-procedural investigations. (*A*) Electrocardiogram showing a right bundle branch block pattern after previous right ventricular outflow tract transannular patch, with QRS duration of 136 ms. (*B*) Doppler assessment of the tricuspid regurgitation jet shows signs of elevated RV systolic pressure. (*C*) Doppler assessment through RPA stent shows significant flow acceleration with a mean pressure gradient of 32 mmHg (slightly underestimated compared to the invasively measured peak-to-peak gradient of 40 mmHg, likely due to echocardiographic assessment of branch PAs in older children being limited due to their anterior position). (*D*) Pre-procedural chest X-ray. PA stents are difficult to visualize due to overlap with the heart shadow. The peripheral pulmonary vasculature seems appropriate, with proximal branches somewhat aneurysmally dilated as seen later in angiograms. (*E*) cMRI assessment of branch PAs is limited due to stent metal artefacts.

At the last clinical review, he was asymptomatic. The physical examination showed a 3/6 systolic murmur at the left upper sternal border. The echocardiography showed signs of raised right ventricular (RV) systolic pressure, estimated at 49 mmHg above right atrial pressure through tricuspid valve regurgitation Doppler assessment (systemic systolic pressure 122 mmHg), and significant flow acceleration across RPA (3.9 m/s) and LPA (could not be reliably measured). Surveillance cardiac magnetic resonance imaging (cMRI) showed RPA:LPA flow distribution ratio was in normal range at 59:41%. The branch PAs were difficult to visualize due to metal artefacts caused by the stents (*Figure 1A–E*).

He was referred for repeat cardiac catheterization to reassess the PA stents in view of significant somatic growth since the last procedure. Due to expected difficult access to the LPA from the femoral access, and expecting bilateral PA interventions, additional jugular access was obtained at the beginning of the procedure, in addition to femoral venous and arterial access. The initial haemodynamic assessment showed the RV systolic pressure was 57% systemic. There was no pulmonary valve stenosis, with free pulmonary insufficiency. There was a 40 mmHg gradient to distal RPA and a 34 mmHg gradient to distal LPA.

**Figure 2:**
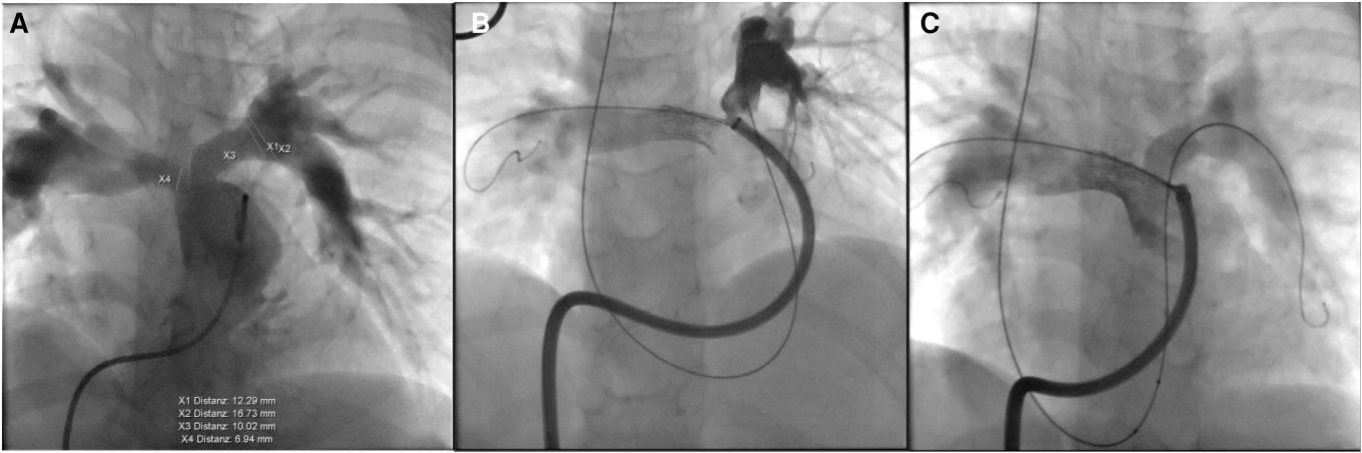
Case 1 angiograms in anteroposterior projection (cranial 30° and left anterior oblique 30°). (*A*) Angiogram in main PA showing severe RPA stenosis as a consequence of the RPA stent integrity loss and mild proximal LPA stenosis. (*B*) After RPA re-stenting with 21 mm AndraStent and dilating it with high-pressure balloon angioplasty in order to achieve complete underlying stent fracture, there is no residual RPA stenosis. (*C*) Final angiogram at PA bifurcation. The LPA stent balloon angioplasty gave a pleasing result without the need to perform re-stenting.

**Figure 3:**
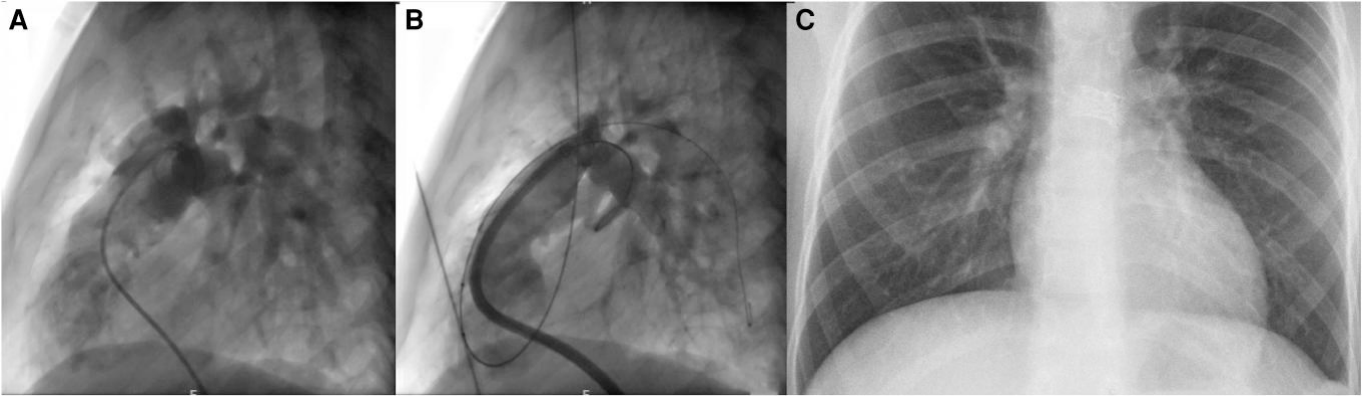
Case 1 angiograms in lateral projection. (*A*) Initial angiogram in main PA. Due to surgically augmented right ventricular outflow tract and aneurysmally dilated peripheral pulmonary arteries, the branch PAs and stents are more difficult to assess in lateral view compared to cranial projections. The lateral projection is however useful for precise balloon and stent positioning, using biplane fluoroscopy with cranial and lateral projection simultaneously. (*B*) Final angiogram in the lateral view helps to confirm absence of vascular wall complications. (*C*) Post-procedural chest X-ray confirming stable PA stents position and absence of lung parenchymal injury.

The angiograms showed that the formerly implanted RPA stent was deformed and partly fractured, offering no further support to the vessel, which caused a severe proximal RPA stenosis (6 mm compared to distal RPA measuring 12 mm). The LPA stent had maintained its integrity and form, although there was intraluminal stenosis present and there was a short length proximal LPA origin stenosis that was not covered by the stent. The stent diameter was 10 mm, while the distal LPA measured 12 mm (*Figures 2A* and *3A*).

Due to severe RPA stenosis, RPA re-stenting was undertaken first. A 0.035 Amplatz extra stiff wire (Cook Medical) was fixed in the right lower PA from the femoral access, and a second Amplatz extra stiff wire was placed into the left lower PA from the jugular vein, in order to secure the access to the vessel in case of jailing with the RPA stent. A 12 Fr long sheath (Cook Medical) was advanced through the RPA stent. A 21 mm AndraStent XXL (Andramed, Reutlingen, Germany) was hand-crimped onto a 12 × 35 mm BiB balloon catheter (NuMED, TX, USA) and positioned within the previous stent, taking care not to cover the PA bifurcation. The stent was deployed slowly under controlled inflation up to 8 atm on the outer balloon (rated burst pressure), showing optimal position afterwards. Due to mild residual stenosis, this was then dilated with a 12 × 20 mm Atlas balloon to 18 atm (rated burst pressure). The final angiogram was pleasing with no residual RPA stenosis and no vascular wall damage (*Figure 2B*).

In view of the good result achieved and a very mild proximal LPA stenosis (the uncovered part), while the LPA stent still had its integrity, we decided to proceed with a high-pressure balloon LPA angioplasty with a 12 × 20 mm Atlas to 18 atm. This showed a good final result on repeat angiogram without a significant residual stenosis, preserved stent integrity and no vascular wall damage (*Figures 2C* and *3B*).

Final haemodynamics showed the RV systolic pressure dropped to 30% systemic, and there were a 4 mmHg gradient and a 2 mmHg gradient from main PA to the RPA and LPA, respectively. A repeat cardiac catheterization is planned in 2–4 years, with likely the need to further dilate the newly implanted RPA stent and possibly re-stent and dilate the LPA stent to match for somatic growth.

### Case 2

The second case is a 10-year-old Caucasian girl, born with truncus arteriosus type A1. She underwent bilateral PA banding at the age of 3 days. At the age of 8 months, a full correction was performed with PA debanding, VSD patch repair, reconstruction of the truncal valve, implantation of a 14 mm Contegra RV-PA conduit, as well as intraoperative hybrid insertion of bilateral PA stents (5 × 12 mm Formula to LPA and 4 × 12 mm Formula to RPA). One year later, further re-stenting to the LPA with 6 × 16 mm Formula was required. She underwent two balloon pulmonary angioplasties to upsize the PA stents in due course, with the last one undertaken at the age of 5 years: both stents were dilated sequentially with high-pressure balloons; final balloon size was 12 mm Atlas to 18 atm.

**Figure 4:**
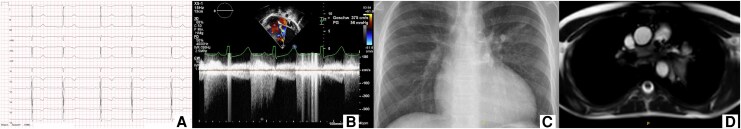
Case 2 pre-procedural investigations. (*A*) Electrocardiogram showing sinus rhythm with normal QRS duration (94 ms). There are repolarization abnormalities with biphasic T waves in precordial leads V4–V5. (*B*) Doppler assessment of the tricuspid regurgitation jet shows signs of elevated RV pressure (possibly underestimated due to incomplete Doppler signal envelope). (*C*) Pre-procedural chest X-ray. The peripheral pulmonary vasculature is well developed to all lung lobes. (*D*) cMRI assessment of branch PAs is again limited due to stent metal artefacts.

At the outpatient review, she reported progressive exercise capacity reduction. The physical examination showed a 3/6 systolic murmur at the left upper sternal border. The echocardiography showed signs of raised RV systolic pressure, estimated at 56 mmHg above right atrial pressure through tricuspid valve regurgitation Doppler assessment (systemic systolic pressure 108 mmHg). The RV-PA conduit and branch PAs could not be assessed. A routine cMRI showed significant bilateral branch PA stenosis, with RPA:LPA flow distribution ratio of 22:78% (*Figure 4A–D*).

**Figure 5:**
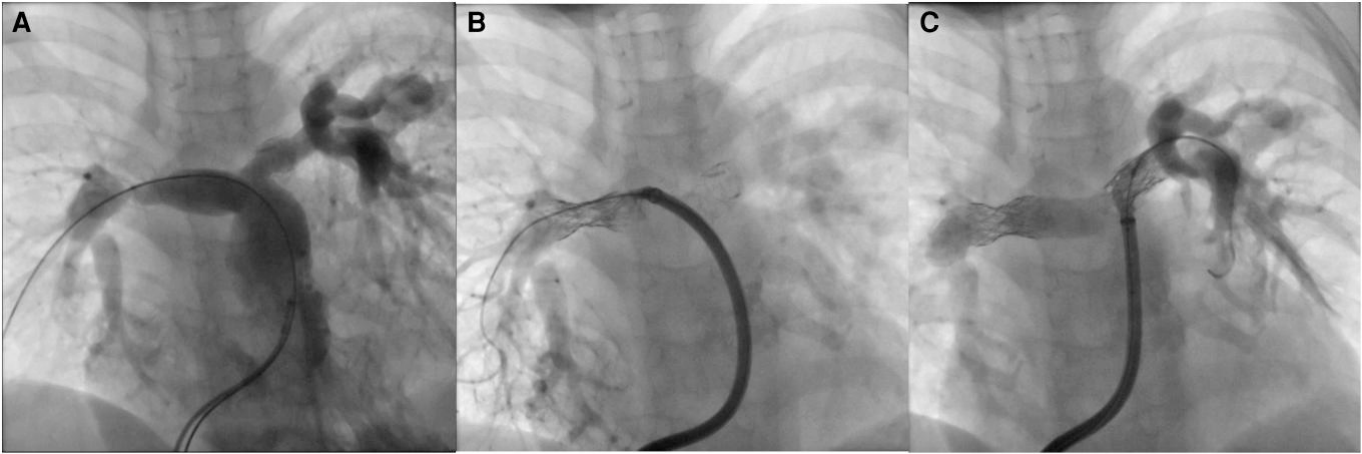
Case 2 angiograms in anteroposterior projection (cranial 30°). (*A*) Angiogram in the aneurysmatically dilated Contegra conduit shows bilateral severe branch PA stenosis, which is especially pronounced on the distal RPA, caused both by the stent integrity loss and intraluminal stenosis. There is a fracture on the proximal LPA stent, which causes proximal LPA stenosis. (*B*) Control angiogram after re-stenting the RPA. There is a significant residual stenosis caused by the previous stent. (*C*) After dilating the RPA stent with high-pressure balloon angioplasty in order to achieve complete fracture of the previously implanted stent. The LPA was re-stented throughout up to bifurcation, with pleasing results.

**Figure 6:**
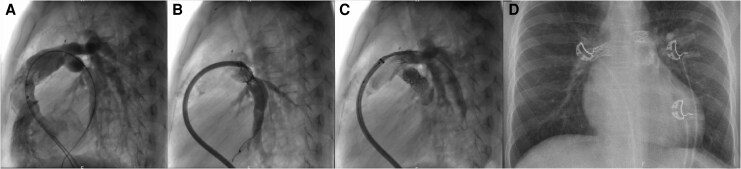
Case 2 angiograms in lateral projection. (*A*) Initial angiogram in the Contegra conduit. The RPA stenosis can be well observed. The LPA is somewhat harder to assess due to overlap. (*B*) Same image as in *Figure 5B* after initial RPA re-stenting before high-pressure balloon angioplasty, in lateral projection. (*C*) Final angiogram in lateral projection confirming resolved bilateral PA stenosis and no obstruction of PA bifurcation by the implanted stents. There is no vascular wall damage. (*D*) Post-procedural chest X-ray confirming unchanged PA stents position and absence of lung parenchymal injury.

She was referred for repeat cardiac catheterization to assess RV pressures and the severity of branch PA stenosis. The haemodynamic assessment showed that RV systolic pressure was 88% systemic. There was a 14 mmHg gradient through the RV-PA conduit. The gradient from main PA to RPA was 38 mmHg and to LPA 28 mmHg. The angiography showed fracture of the RPA stent with loss of its integrity and severe intraluminal stenosis with poor distal RPA perfusion. Distal RPA was good size and measured 12 mm. The LPA had complete proximal stent fracture resulting in proximal LPA stenosis immediately after PA bifurcation. Distal stent had significant intraluminal stenosis as a result of previous oversized balloon dilatation (the distal vessel was 10 mm, while the stent diameter measured 12 mm) (*Figures 5A* and *6A*).

In view of almost systemic RV pressures as a result of severe bilateral branch PA stenosis, recruitment of both RPA and LPA was undertaken. We focused on RPA first due to the severity of the lesion. A 0.035 Amplatz extra stiff wire was fixed in the right lower PA, and a 12 Fr long sheath (Cook Medical) was advanced through the stent. A 21 mm AndraStent XXL was hand-crimped onto a 12 × 45 mm BiB and carefully positioned within the previous RPA stent, taking care not to cover any of the peripheral branches. The stent was deployed with slow careful inflation to achieve optimal position and postdilated with 12 × 20 mm Atlas due to severe stenosis caused by the previous stent (*Figures 5B* and *6B*); at 18 atm, the stenosis was abolished and the stent opened well with no residual gradient through RPA and no vascular wall damage.

The Amplatz extra stiff wire was then re-positioned into the left lower PA, followed by crossing through the LPA stent with the 12Fr long sheath. A 21 mm AndraStent XXL on 10 × 35 mm BiB was positioned to cover both proximal stenosis as well as the distal part of the previous stent, without covering the peripheral branches. The stent was deployed carefully with great stability and complete expansion at rated burst pressure of the outer balloon (9 atm). There was no residual stenosis visually on the newly implanted stent, it opposed the vessel walls well and there was no vascular damage. It also matched completely the distal vessel diameter, and therefore, no further high-pressure balloon dilatation was undertaken even though a small gradient across LPA persisted (7 mmHg without an obvious anatomical substrate) (*Figures 5C* and *6C*).

Final haemodynamics showed a pleasing result with RV pressure now 50% systemic. The implanted AndraStents will require further dilation in adolescence after full somatic growth has taken place.

## Discussion

Implantation of small-sized stents in infants has significantly improved treatment options for branch PA stenosis in this patient population, especially as surgical branch PA plasty attempts frequently leads to re-stenosis in the mid- and long-term follow-up,^6^ likely due to anatomical factors such as fibrose tissue, the use of patch material, geometrical distortion, or compression by the surrounding structures. On the other hand, the implanted stents require serial balloon dilation, with limited maximal dimensions. Percutaneous treatment of branch PA stenosis due to undersized previously implanted stents that cannot be expanded to match the patient’s somatic growth is challenging, and some authors consider small-sized stent implantation to be a palliative treatment option until further surgical treatment with stent removal and extensive PA plasty is undertaken.^7^ However, transcatheter definitive treatment can be achieved in many patients with excellent results when the case is planned in detail beforehand.

High-pressure balloon dilatation of the implanted stents above their labelled maximal diameter will inevitably result in stent fracture.^8^ Furthermore, potential for stent foreshortening needs to be taken into consideration: the closed-cell stents experience more foreshortening than open-cell stents and are more resistant to fracture compared to open-cell stents.^9,10^ All types of stents can be opened with high-pressure balloons, as has been demonstrated with benchside testing.^8,10,11^ In our two patients, one had open-cell type stents (Valeo) and one a hybrid type (Formula).

The most important steps to consider when dealing with patients after implantation of a small-sized stents in branch PAs are the following:

1. Serial balloon angioplasty with incremental (2 mm) balloon sizes results in a more stable stent expansion and complete fracture, especially in stents that foreshorten significantly (closed-cell) and where direct large diameter dilation of stents can result in ‘napkin-ring’ configuration which can be more resistant to fracture.^10,11^ Neither of our patients experienced significant stents foreshortening during previous balloon angioplasties, both due to the stent design (open-cell/hybrid type) and due to sequential stent upsizing.
2. When approaching the fracture size, consideration of second stent implantation within the initial stent before stent fracture is of utmost importance, as it improves maintenance of vessel patency, improves endothelialization rates, stabilizes the fractured stent, and avoids protrusion of struts inside the vessel lumen.^10,12^ This is very well demonstrated in both of our patients, where dilating the stents over the expandability limit with high-pressure balloons in previous catheterization lead to subsequent vessel collapse with severe residual stenosis. Therefore, a calculated approach with planned re-stenting with adult-sized stents when approaching the maximal expansion diameters, followed by intentional fracture of the initial stent with high-pressure balloons, is highly advisable in our opinion and has been suggested as the integral part of the treatment strategy by some other groups.^10^ However, this is still controversial and considered as unnecessary by some authors.^13^
3. The timing of the re-stenting with adult-sized stents will be determined not only by the anatomy but also by the technical aspects, as these require large delivery sheaths and are therefore challenging in small size patients. We chose the AndraStent XXL (hybrid type stent) due to its minimal foreshortening characteristics even with large balloon diameters, in order to avoid re-stenosis due to insufficient vessel coverage after future stent re-dilations. The balloon choice was determined by the balloon type availability in our cardiac catheterization laboratory, as well as by the perceived benefit of controllable stent deployment on BiB.
4. The choice of the stent type (covered on uncovered) is a topic for discussion. There is still limited experience and literature on the topic of intentional stent fracture. The current experience suggests no significant difference in the major complications, such as vascular wall damage, in stent re-dilation with or without fracturing.^5,10,13^ We have chosen to use uncovered stents in our cases accordingly, allowing for smaller delivery sheath size, as well as not to obstruct peripheral PA branches in case of unintentional distal stent displacement. The availability of a covered stent in the catheterization laboratory as an emergency bail-out option in case of vascular perforation is however advisable, just as in all cases of stent angioplasty.

## Patient’s perspective

With the expanding experience with intentional stent fracture to recruit branch PAs after small-sized stent implantation in infancy, we are hoping that the interventional society will be able to develop clearer algorithms as to what the optimal and safest approach is to achieve best and sustainable results up to adulthood. This would avoid the need for more invasive treatments, such as surgical stent removal and PA plasty, especially as the latter can ultimately lead to further need for percutaneous stent angioplasty.

Branch PA stenting carries a risk of serious adverse events (SAE) inherent to the procedure, such as development of PA aneurysm or dissection, stent embolization, vascular access complications, and peripheral pulmonary arteries damage with the wire. The CRISP score (Catheterization RISk score for Pediatrics)^14^ helps to predict the occurrence of a SAE for individual patients undergoing paediatric cardiac catheterization procedures and is helpful when considering the risk-benefit ratio in comparison to surgical management. Using the CRISP score calculation algorithm, the presented procedures fall into CRISP Category 2, which predicts a relatively low 2.6% risk of SAE. Neither of the two patients suffered a procedure-related complication during the current or previous cardiac catheterization.

Another important aspect to consider is the cumulative patient's procedural radiation exposure with multiple cardiac catheterizations. The dose area product in the presented cases was 5025 cGy × cm2 for Patient 1 (37 kg) and 2242.3 cGy × cm2 for Patient 2 (25 kg), which taking into account the complexity of the procedure is comparable to previously reported patient radiation doses in therapeutic procedures in paediatric interventional cardiology.^15^

## Conclusion

Percutaneous treatment of branch PA stenosis in small children has advanced significantly; the paediatric interventional cardiologist now needs to focus on the older patient where the implanted stents have reached the limit of expandability. Rather than considering the initial procedure as a palliative interim option, we need to develop a lifelong treatment strategy. With emerging experience from other groups, and practical cases reported, the knowledge and confidence to recruit branch PAs in cardiac catheterization laboratory will grow accordingly.

## Data Availability

All available data are presented within the manuscript.

## Lead author biography

Ines Hribernik is a paediatric and congenital interventional cardiologist, trained in Leeds (UK). After finishing her training, she worked as a consultant paediatric interventional cardiologist in Bristol (UK) and Barcelona (Spain) and is currently working as the lead of the paediatric interventional cardiology programme in University Hospital Muenster. Her main interests are neonatal percutaneous palliations and complex interventions in congenital heart disease.

## Notes

**Consent:** Informed consent by the patients’ family has been obtained for this report in accordance with COPE guidelines.
